# Physical Activity in Crisis: The Impact of COVID-19 on Danes' Physical Activity Behavior

**DOI:** 10.3389/fspor.2020.610255

**Published:** 2021-02-01

**Authors:** Tanja Schmidt, Charlotte Skau Pawlowski

**Affiliations:** Research Unit for Active Living, Department of Sports Sciences and Clinical Biomechanics, University of Southern Denmark, Odense, Denmark

**Keywords:** coronavirus, leisure physical activity, teens, younger adults, adults, older adults

## Abstract

**Objectives:** Because of the COVID-19 pandemic, societies have been shut down, changing the lives of citizens worldwide, including their physical activity (PA) behavior. This cross-sectional study aimed to investigate the impact of the shutdown on Danish citizens' leisure PA throughout different stages of life: 15–18, 19–29, 30–59, and 60+ years.

**Methods:** Between April 3 and 15, 2020, while Denmark was shut down, a survey was distributed through online platforms. Danish citizens (>15 years) could participate in the study, answering questions about their PA behavior before, and during the shutdown.

**Results:** The number of total participants was 1,802; 7.9% teens, 21.5% younger adults, 58.7% adults, and 11.9% older adults. Mean minutes of PA decreased 16.1% from before to during the shutdown. Teens had the largest decrease in PA (36.6%) followed by older (24.9%) and younger adults (21.3%). Low educated (31.5%) and those living in rural areas (30.9%) experienced the largest decrease in PA. Main factors for not doing PA during the shutdown were that they missed what they used to do, lacked social support, and did not have access to the right facilities.

**Conclusions:** During the shutdown, the Danish population struggled even more to comply with national PA guidelines compared with before the shutdown. Although social distancing and shutting down sports facilities are important for preventing the spread of the virus, it can have negative consequences for teens' and younger and older adults' opportunities and motivation for PA, leading to an alarming decrease in PA, and, consequently, will have major public health consequences.

## Introduction

On March 11, 2020, the World Health Organization declared the coronavirus disease 2019 (COVID-19) to be a pandemic (World Health Organization, 2020). As of December 11, 2020, Johns Hopkins University reported 101,027 confirmed cases of COVID-19 and 918 deaths in Denmark (University, 2020). Since March 11, 2020, Denmark was shut down as a basic means of limiting people's exposure to the virus, including closures of schools, workplaces, fitness centers, and sport associations, and social gatherings of more than 10 people (Ministry, 2020). These decisions will inevitably affect people's regular leisure physical activity (PA) (Hall et al., 2020). In Denmark, PA plays an important role in many people's everyday life and is often carried out as organized sport in sports associations or fitness centers (Pilgaard and Rask, 2016). The beneficial effects of regular PA on many health outcomes are well-established (Powell et al., 2019). PA has an important role in reducing impact of the COVID-19 pandemic by having a positive effect on immunity (Jones and Davison, 2019), inflammation (Miles et al., 2019), and viral respiratory infections (Nieman and Wentz, 2019). PA can also reduce stress and depression (Powell et al., 2019), which may increase during the pandemic due to health threats, job loss, reduced income, and isolation from social contacts (Douglas et al., 2020). Due to the multiple benefits of PA, it is important to sustain an active lifestyle during the COVID-19 shutdown. Nevertheless, the world has struggled with a physical inactivity pandemic for several years (Kohl et al., 2012), affecting especially those from lower socioeconomic groups (Pilgaard and Rask, 2016) and younger (Rasmussen et al., 2019) and older adults (Bauman et al., 2016). It is argued that the COVID-19 pandemic will increase those struggles and persist long after the world has recovered from the pandemic (Hall et al., 2020).

Several studies from different European, Asian, and American countries have—to some degree—investigated the impact of the COVID-19 shutdown on PA behavior (Cheval et al., 2020; López-Bueno et al., 2020; Maugeri et al., 2020; Ong et al., 2020; Rhodes et al., 2020; Rogers et al., 2020; Smith et al., 2020; Yamada et al., 2020). All reported some form of decrease in PA from before to during the shutdown, whereas three of the studies also reported no change (Rogers et al., 2020; Smith et al., 2020) or an increase in PA (Cheval et al., 2020) for some specific groups. This may be due to poor measures of PA and small sample sizes as two of the studies did not report a specific PA measurement tool used (Rogers et al., 2020; Smith et al., 2020), and one of the studies only included 267 participants (Cheval et al., 2020). Seven of the studies were cross-sectional, using retrospective and self-reported online surveys to measure PA, whereas one was a longitudinal study that used device-based PA monitoring (Ong et al., 2020). Some studies assessed the impact of the shutdown on PA for different age groups (Maugeri et al., 2020; Rogers et al., 2020), whereas one study only focused on older adults (Yamada et al., 2020). None of the studies seem to have included people younger than 18 years old. None of the studies were conducted in Denmark or any other Scandinavian country.

The lack of Scandinavian studies and studies on specific age groups suggest a gap in the literature. This gap is important to fill to understand the worldwide consequences of the pandemic on PA behavior as well as the specific consequences for the Danish population. This supports political leaders in their decision making on COVID-19–related restrictions and helps public health officials to make recommendations for PA during a shutdown. The aim of this study was to investigate the impact of the shutdown on Danish citizens' leisure PA throughout different stages of life. This is done by analyzing retrospective and self-reported data on PA behavior before and during the COVID-19 shutdown among teens, younger adults, adults, and older adults.

## Methods

### Study Design and Recruitment

The present paper is a cross-sectional study conducted in Denmark between April 3 and 15, 2020, during the COVID-19 shutdown. April 15 was chosen as the end date for the study because several restrictions from the Danish government were eased from that day.

On April 3, 2020, the study announced a press release, including a link to an online survey to recruit participants. The survey was also distributed through Facebook by posting a link to the survey on the university department's Facebook site and afterward sharing that link in different Facebook groups, on LinkedIn, and through the university's work mail. The Facebook post was boosted 1 time after 1 week to gain further reach. The study was advertised as a survey on people's movement behavior during the COVID-19 shutdown. It was explicitly mentioned that everyone, no matter how much or how little previous or current PA experience they had, could participate in the study. Inclusion criteria were people over the age of 15 years.

### Data Collection

The online survey was created in SurveyXact based on a standardized Danish national PA survey repeated every fourth year (Pilgaard and Rask, 2016), which asks citizens about their PA behavior at work/school, during leisure time, and about active transportation. For this study, only questions about leisure PA were assessed. The survey started and ended with a range of background information on age, gender, education, ethnicity, job situation, and living situation (living in a small or large city, alone or with family). This was followed by a range of questions on the participants' general leisure PA behavior before and during the COVID-19 shutdown: “How much time did you usually spend on PA per week before the shutdown?” “How much time per week do you spend during the shutdown on PA (please write the number of minutes in total for 1 week)?” “How often were you physically active before the shutdown?” “How often are you physically active during the shutdown (5 or more times per week, 2–4 times per week, once a week, 2–3 times a month, rarely/never)?” It was clearly stated not to include active transportation, housework, or strenuous work. The participants were also asked, “What types of PA have you done on a regular basis during the last 12 months before the shutdown/during the shutdown?” The survey included examples of leisure PA (e.g., team ball games, outdoor activities, and gymnastics). Other questions were “Where have you primarily been physically active during the last 12 months before the shutdown/during the shutdown (e.g., sport associations, fitness/private center, at home, in public)?” “Who where you the most physically active with before and during the shutdown (alone, with friends, with colleagues, with family, with people I don't know)?” “Have you gained new PA habits during the shutdown?” Last, the participants were asked to identify perceived factors important for whether they are physically active during the shutdown to assess contextual factors that may explain a possible change in PA behavior. All questions came from the original national PA survey and were adapted to the specific COVID-19 shutdown.

The survey took, on average, 10 min to complete. The survey was tested on different age groups prior to its release, to ensure that the adaptions were understandable. The survey was found understandable by all age groups, but feedback from the test respondents resulted in the inclusion of additional response categories to the question on important factors for PA. The survey was only available in Danish.

### Ethical Approval

Participants were given information about the study in the introduction to the survey. At the end of the survey, participants were able to read additional information on (1) how data were used and stored, (2) that they accepted that their data would be used for research purposes upon completion of the survey, and (3) that they were able to withdraw from the study at any time. The study and its data-management procedures were ethically approved by the Research & Innovation Organization of the University of Southern Denmark (10.975).

### Analysis

Data were downloaded from SurveyXact and uploaded in IBM SPSS Statistics version 25. Paired *t*-tests were performed to test for significant overall changes in weekly minutes of PA between, before, and during the shutdown as well as PA changes for several demographic groups (e.g., gender differences or differences in PA by education) between, before, and during the shutdown (Table 2). Pearson Chi-squared tests were performed for specific subgroup analyses on the four age groups: teens (15–18 years), young adults (19–29 years), adults (30–59 years), and older adults (60+ years) to test for independence at a 0.05 significance level between the four age groups before and during the shutdown (Table 3).

## Results

### Overview

A total of 1,347 participants answered the full survey, and an additional 455 partly answered the survey. All 1,802 participants were included in the analysis. Participants' demographics are presented in Table 1. The oldest participant was 85 years old, and the youngest was 15 years old. Mainly females (75.8%) and Danish-speaking citizens (98.3%) participated in the survey. Two thirds of all participants (74.5%) lived in large towns and mainly worked at home during the shutdown (43.4%).

**Table 1:** Descriptive overview of participants' demographics in total and for the four age groups.

		**15–18 years old**	**19–29 years old**	**30–59 years old**	**60+ years old**	**TOTAL**
Age total		142 (7.9%)	388 (21.5%)	1,057 (58.7%)	215 (11.9%)	1,802 (100%)
Gender	Male	28 (19.7%)	115 (29.6%)	244 (23.1%)	46 (21.6%)	433 (24.1%)
	Female	113 (79.6%)	273 (70.4%)	810 (76.8%)	167 (78.4%)	1,363 (75.8%)
	Other	1 (0.7%)	0 (0.0%)	1 (0.1%)	0 (0.0%)	2 (0.1%)
Primary language at home	Danish	136 (96.5%)	385 (99.2%)	1,036 (98.2%)	209 (98.1%)	1,766 (98.3%)
	Other languages	5 (3.5%)	3 (0.8%)	19 (1.8%)	4 (1.9%)	31 (1.7%)
Education	Higher education under 3 years (e.g., pharmacist, craftsman)	80 (96.4%)	125 (44.2%)	149 (17.6%)	65 (40.1%)	419 (30.4%)
	Middle higher education 3–4 years (e.g., nurse, schoolteacher)	0 (0.0%)	54 (19.1%)	282 (33.2%)	59 (36.4%)	395 (28.7%)
	Higher education (including any bachelor's degree) over 4 years (e.g., doctor, MSc)	0 (0.0%)	99 (35.0%)	409 (48.2%)	34 (21.0%)	542 (39.4%)
	Other	3 (3.6%)	5 (1.8%)	9 (1.1%)	4 (2.5%)	21 (1.5%)
Region	Large city (>15,000 citizens)	42 (50.6%)	249 (88.0%)	611 (72.1%)	122 (75.3%)	1,024 (74.5%)
	Town (2,000–15,000 citizens)	25 (30.1%)	23 (8.1%)	158 (18.7%)	20 (12.3%)	226 (16.4%)
	Village or countryside (<2,000 citizens)	16 (19.3%)	11 (3.9%)	78 (9.2%)	20 (12.3%)	125 (9.1%)
Living situation	Live alone	0 (0.0%)	62 (22.0%)	95 (11.2%)	46 (28.4%)	203 (14.8%)
	Live with one or more children	0 (0.0%)	4 (1.4%)	85 (10.0%)	3 (1.9%)	92 (6.7%)
	Live with my partner	1 (1.2%)	118 (41.8%)	134 (15.8%)	101 (62.3%)	354 (25.8%)
	Live with my partner and children	0 (0.0%)	16 (5.7%)	513 (60.6%)	8 (4.9%)	537 (39.1%)
	Live with my parents	78 (94.0%)	29 (10.3%)	5 (0.6%)	0 (0.0%)	112 (8.2%)
	Other	4 (4.8%)	53 (18.8%)	14 (1.7%)	4 (2.5%)	75 (5.5%)
Job situation during the COVID-19 shutdown	Work outside of home	5 (3.7%)	24 (6.3%)	161 (15.3%)	20 (9.6%)	210 (11.7%)
	Work at home	5 (3.7%)	77 (20.4%)	657 (62.6%)	43 (20.6%)	782 (43.4%)
	Work partly outside partly at home	4 (3.0%)	4 (1.1%)	20 (1.9%)	2 (1.0%)	30 (1.7%)
	Do not work	6 (4.4%)	55 (14.6%)	135 (12.9%)	134 (64.1%)	330 (18.3%)
	Sent home student	115 (85.2%)	213 (56.3%)	28 (2.7%)	1 (0.5%)	357 (19.8%)
	Other	0 (0.0%)	5 (1.3%)	48 (4.6%)	9 (4.3%)	62 (3.4%)

*%, percent*.

Table 2 displays demographic differences in mean minutes of PA per week before and during the shutdown. Overall mean minutes of PA dropped 16.1% (*p* < 0.001) from before to during the shutdown. Of the 215 participants who did not do PA before the shutdown, 51.2% started doing PA during the shutdown, and 7.1% of those doing PA before the shutdown stopped doing PA during the shutdown. Comparing participants' educational level, the highest drop in PA was found among those with the lowest education (−31.5%, *p* < 0.001), whereas the smallest drop was registered among those with the highest education (−1.5%) although it was not significant. Those living in a large city had the smallest decrease in PA (−13.5%, *p* < 0.001) compared with those living in a village or countryside (−30.9%).

**Table 2:** Changes in mean minutes of physical activity from before to during COVID-19 shutdown.

		**Mean minutes of PA per week**		
		**Before (SD)**	**During (SD)**	**Change (percent)**
Physical activity		226.7 (313.9)	190.3 (235.6)	−16.1%^**^
Age	15–18	458.9 (503.5)	290.7 (410.2)	−36.6%^**^
	19–29	281.7 (230.1)	221.8 (264.5)	−21.3%^*^
	30–59	205.2 (344.3)	192.4 (213.5)	−6.3%
	60+	180.4 (181.4)	135.4 (194.1)	−24.9%^**^
Gender	Male	245.8 (264.8)	207.1 (277.5)	−15.7%
	Female	220.1 (328.4)	184.7 (184.7)	−16.1%^*^
Education	Higher education, under 3 years (e.g., secondary education, craftsman, pharmacist)	276.5 (311.9)	189.4 (291.7)	−31.5%^**^
	Middle higher education, 3–4 years (e.g., nurse, physio therapist, schoolteacher)	225.7 (461.9)	188.5 (217.8)	−16.5%
	Higher education (including any bachelor's degree) over 4 years (e.g., doctor, MSc)	196.3 (169.2)	193.4 (206.0)	−1.5%
	Other	209.5 (204.6)	182.7 (158.9)	−12.7%
Job situation during the COVID-19 shutdown	Work outside of home	198.2 (196.8)	207.2 (276.2)	+4.3%
	Work at home	194.1 (301.2)	191.5 (197.9)	−1.3%
	Work partly at home partly outside of home	238.9 (187.4)	151.4 (155.6)	−36.6%^*^
	Do not work	251.9 (411.2)	175.6 (200.7)	−30.3%^*^
	Sent home student	353.2 (362.0)	247.9 (343.9)	−29.8%^**^
Region	Large city (>15,000 citizens)	224.9 (256.5)	194.6 (235.8)	−13.5%^**^
	Town (2,000–15,000 citizens)	227.9 (457.4)	179.2 (178.5)	−21.4%
	Village or countryside (<2,000 citizens)	258.8 (467.4)	178.9 (320.2)	−30.9%
Living situation	Live alone	237.1 (247.2)	218.2 (335.1)	−8.0%
	Live with one or more children	208.7 (249.0)	189.4 (204.9)	−9.3%
	Live with my partner	233.6 (277.0)	179.6 (188.0)	−23.1%^**^
	Live with my partner and one or more children	180.4 (344.7)	171.7 (170.1)	−4.8%
	Live with my parent(s)	414.4 (419.4)	247.1 (337.4)	−40.4%^**^

*Paired t-test. Data presented as mean and percentage of change. PA, physical activity; SD, Standard Deviation*.

** *P < 0.001*,

* *P < 0.05*.

### Age Specific Differences

Table 3 presents additional analyses made for the four specific age groups.

**Table 3:** Differences in physical activity for the four age groups before and during shutdown.

		**BEFORE**				**DURING**			
		**15–18 years**	**19–29 years**	**30–59 years**	**60+ years**	**15–18 years**	**18–29 years**	**30–59 years**	**60+ years**
Are you in general physically active	Yes	88.0%	90.9%	87.3%	83.4%	83.2%	88.2%	87.5%	83.3%
How often where/are you physically active	5 times per week or more	36.3%^**^	25.0%^**^	17.5%^**^	20.5%^**^	44.9%^**^	27.5%^**^	31.4%^**^	25.9%^**^
	2–4 times per week	50.4%^**^	59.4%^**^	61.4%^**^	45.3%^**^	34.6%^**^	57.0%^**^	49.2%^**^	28.0%^**^
	1 time per week	9.7%^**^	6.9%^**^	12.0%^**^	15.5%^**^	11.5%^**^	7.9%^**^	8.1%^**^	5.6%^**^
	2–3 times a month	1.8%^**^	4.7%^**^	2.7%^**^	8.1%^**^	2.6%^**^	2.3%^**^	1.2%^**^	3.5%^**^
	Rarely/never	1.8%^**^	4.1%^**^	6.3%^**^	10.6%^**^	6.4%^**^	5.3%^**^	10.2%^**^	37.1%^**^
What types of PA have you done on a regular basis during the last 12 months before/during the shutdown	Team ball games (e.g., soccer, handball)	28.9%^**^	23.5%^**^	10.6%^**^	3.7%^**^	7.0%^**^	1.3%^**^	1.8%^**^	0.0%^**^
	Individual ball games (e.g., tennis, golf)	19.0%^**^	10.6%^**^	7.6%^**^	7.4%^**^	4.2%^*^	0.3%^*^	2.1%^*^	1.9%^*^
	Outdoor activities (e.g., hiking, cycling)	51.4%^**^	61.9%^**^	70.9%^**^	62.8%^**^	52.1%^**^	63.4%^**^	69.3%^**^	63.7%^**^
	Water activities (e.g., swimming, kayak)	12.7%^*^	12.4%^*^	13.2%^*^	20.0%^*^	4.2%	1.8%	1.8%	0.5%
	Street activities (e.g., CrossFit, skateboard)	30.3%^**^	52.1%^**^	30.7%^**^	19.1%^**^	25.4%^**^	36.3%^**^	17.0%^**^	3.7%^**^
	Gymnastics etc. (e.g., dance, yoga)	23.9%^**^	18.3%^**^	23.6%^**^	32.6%^**^	11.3%^*^	12.4%^*^	17.5%^*^	21.4%^*^
	Other activity (e.g., fishing, scout)	9.2%^*^	8.8%^*^	5.5%^*^	1.9%^*^	2.1%	4.9%	3.0%	2.3%
Where have you primarily been physically active during the last 12 months before/during the shutdown	Sport associations	53.0%^**^	31.8%^**^	23.2%^**^	30.5%^**^	4.8%^**^	0.4%^**^	0.4%^**^	0.7%^**^
	Fitness/private center	21.4%^**^	42.9%^**^	31.0%^**^	20.4%^**^	0.0%^**^	1.5%^**^	0.4%^**^	0.0%^**^
	At work/youth club	3.4%^**^	3.3%^**^	2.2%^**^	3.6%^**^	0.0%^**^	0.4%^**^	0.4%^**^	0.7%^**^
	At home	7.7%^**^	2.1%^**^	4.4%	6.6%	57.1%^**^	42.2%^**^	28.0%^**^	29.2%^**^
	In public	8.5%^**^	15.2%^**^	34.2%^**^	33.5%^**^	33.3%^**^	51.5%^**^	66.7%^**^	68.8%^**^
	Other	6.0%^**^	4.8%^**^	4.9%^**^	5.4%^**^	4.8%^**^	4.1%^**^	4.1%^**^	0.7%^**^
Who where you the most physically active with	Alone	10.6%^**^	29.4%^**^	37.9%^**^	28.2%^**^	57.7%^**^	61.1%^**^	46.6%^**^	56.7%^**^
	With friends	83.5%^**^	55.9%^**^	38.6%^**^	40.8%^**^	1.4%^**^	11.1%^**^	8.2%^**^	8.2%^**^
	With colleagues	1.2%^**^	4.0%^**^	3.3%^**^	3.5%^**^	0.0%^**^	0.4%^**^	0.8%^**^	0.0%^**^
	With family	4.7%^**^	6.3%^**^	11%^**^	15%^**^	40.8%^**^	27.4%^**^	44.4%^**^	35.1%^**^
	With people I don't know	0.0%^**^	4.4%^**^	9.0%^**^	12.7%^**^	0.0%^**^	0.0%^**^	0.0%^**^	0.0%^**^
Have you gained new PA habits during the shutdown	Yes, do other PA					28.9%^**^	31.2%^**^	32.5%^**^	15.8%^**^
	Yes, do the same PA but in a different way					19.7%^*^	31.7%^*^	26.9%^*^	20.0%^*^
	No					7.7%^**^	10.6%^**^	20.7%^**^	32.1%^**^
Which factors are important for whether you are physically active during the shutdown	None					8.5%	10.8%^**^	26.4%^**^	27.0%^**^
	I lack time/use time on something else					12.0%	5.9%^**^	12.7%^**^	4.2%^**^
	I don't feel like it					14.8%	14.2%^**^	6.8%^**^	6.0%^**^
	I don't feel safe going outside my home					4.2%	4.4%^**^	3.4%^**^	11.6%^**^
	I lack someone to do PA with					28.9%	26.0%^**^	17.7%^**^	16.3%^**^
	I don't have access to the right facilities					28.9%	41.0%^**^	28.9%^**^	27.9%^**^
	I miss what I used to do					33.1%	42.5%^**^	28.0%^**^	33.0%^**^
	I have been sick/in quarantine					3.5%	4.4%	2.3%	2.8%
	Other					4.2%	5.7%	7.5%	5.6%

*Pearson Chi-squared test for independence between the four age groups. PA, physical activity; %, percent*.

** *P < 0.001*,

* *P < 0.05*.

#### Teens

Teens experienced the largest decrease in mean minutes of PA per week (36.6%) (Table 2). Those who did PA 5 times per week or more increased by 19.1% during the shutdown, whereas those who rarely or never did PA increased by 71.8%. Before the shutdown, more than half of the teens did outdoor activities, and almost a third did street activities and team ball games, primarily in sports associations (53.0%). During the shutdown, more than half still did outdoor activities, but only a fourth did street activities, and only 7.0% did team ball games. Activities during the shutdown were primarily done at home (57.1%) or in public (33.3%). Before the shutdown, PA was mostly done with friends but was during the shutdown mostly done alone or with family. Teens perceived most factors important for whether they are physically active during the shutdown. Only 8.5% identified no factors.

#### Younger Adults

Younger adults experienced a decrease of 21.3% in mean minutes of PA per week (Table 2). Younger adults who did PA 5 times per week or more increased 9.1% during the shutdown, and those who rarely or never did PA increased 22.6% during the shutdown. Primary activities before the shutdown were outdoor activities, street activities, and team ball games. During the shutdown, team ball games dropped to 1.3%, whereas 63.4% did outdoor activities. Before the shutdown, PA was mostly done with friends or alone, in sport associations or fitness centers, whereas, during the shutdown, it was mostly done alone in public and at home. The greatest factors important for whether they were physically active during the shutdown were that they missed what they used to do (42.5%) and felt that they did not have access to the right facilities (41.0%).

#### Adults

Adults experienced the smallest decrease in mean minutes of PA per week (6.3%) (Table 2). Those doing PA 5 times or more per week increased by 44.3%, and those who rarely or never did PA increased by 38.2%. Adults mostly did outdoor PA before and during the shutdown. PA was done in public (34.2%) or fitness centers (31.0%) before the shutdown and was done in public (66.7%) and at home (28.0%) during the shutdown. Most adults did PA alone or with friends before the shutdown. During the shutdown, most adults were still doing PA alone, whereas almost half did PA with their family. Those who lived with their partner and children experienced the smallest decrease in minutes of PA per week (4.8%), whereas those living with their partner (without children) experienced the largest decrease (40.4%) (Table 2). Finally, 26.4% of the adults did not identify any factors important for PA, but almost every third respondent missed what they used to do or felt that they did not have access to the right facilities.

#### Older Adults

Older adults experienced a significant decrease in mean minutes of PA per week (24.9%) (Table 2), and over a third were rarely or never physically active during the shutdown. Before the shutdown, older adults primarily did outdoor activities and a third did gymnastics in sports associations (30.5%) and fitness or private centers (20.4%). During the shutdown, most older adults did outdoor activities such as walking in public and gymnastics at home. Before the shutdown, PA was primarily done with friends (40.8%) and alone (28.2%). During the shutdown, more than half of older adults did PA alone, and over a third did PA with family members. Furthermore, a third of the participating older adults stated that they did not gain any new PA habits during the shutdown, 33.0% said that they missed what they used to do, and 27.9% felt that they did not have access to the right facilities.

## Discussion

The study revealed a decrease in mean minutes of PA by 16.1% from before to during the COVID-19 shutdown. This finding is worrying given the beneficial effects of regular PA on many health outcomes and its important role in reducing the impact of the COVID-19 pandemic (Powell et al., 2019). It is particularly alarming that 7.1% of participants, who did PA before, stopped doing PA during the shutdown. One study by Olsen et al. (2008) finds that only 2 weeks of a marked reduction in PA among healthy individuals had several crucial health consequences. Although it is difficult to predict the long-term health implications due to decreased PA following an pandemic such as COVID-19, previous research on the 2011 earthquake and tsunami devastating East Japan reports a lasting decrease in PA over 3 years following the disaster (Okazaki et al., 2015). This suggests that the COVID-19 shutdown might have long-term consequences for peoples' PA.

Looking at the different subgroups, the drop in PA was even higher. The lower-educated respondents and those living on the countryside significantly decreased their mean minutes of PA per week more than other subgroups. These findings are similar to other studies on PA behavior during the COVID-19 pandemic (López-Bueno et al., 2020; Rhodes et al., 2020). Unfortunately, low-educated individuals and residents living in rural areas are those who already are least likely to meet PA recommendations both inside (Pilgaard and Rask, 2016) and outside of Denmark (Parks et al., 2003) and, therefore, should be given special priority in public health promotion during and after the COVID-19 shutdown. The study also found a significant disparity in PA behavior among the different age groups, which is supported by previous studies on PA behavior during the COVID-19 pandemic (Cheval et al., 2020; López-Bueno et al., 2020; Maugeri et al., 2020; Ong et al., 2020; Rhodes et al., 2020; Rogers et al., 2020; Yamada et al., 2020) and discussed in the following.

### Teens

Teens experienced the largest decrease in mean minutes of PA (36.6%). At the same time, there was a substantial increase in individuals who rarely or never did PA during the shutdown (71.9%). Not doing PA for a longer time in this stage of life is alarming as this is the time when individuals begin to formulate their healthy habits for life (Telama, 2009). A study by Maugeri et al. (2020) finds similar results. They use an adapted version of the IPAQ questionnaire on a sample of 2,524 individuals and find a 34.4% decrease in mean METs min/week for younger participants (<21 years old) (Maugeri et al., 2020). The age range of the <21 age category is, however, not listed in the study, and it is, therefore, unknown whether they included 15–18-year-old adults. The teens were most often active in sports associations together with friends before the shutdown. When these facilities were closed, more than half did PA at home and alone. In fact, a third missed what they used to do, felt that they did not have access to the right facilities, and lacked someone with whom to do PA. For young people, social interactions with friends significantly influences PA behavior (Macdonald-Wallis et al., 2012), a factor that may have been negatively impacted by social distancing mandates as a result of COVID-19.

### Younger Adults

Younger adults had a decrease of 21.3% in mean minutes of PA per week from before to during the shutdown. This decrease may be due to their limited abilities to perform their usual PA routines. The younger adults were not able to do their usual PA in sport associations and fitness centers together with friends during the shutdown. Moreover, almost half of the younger adults reported that they missed what they used to do and felt that they did not have access to the right facilities. This suggests that restricting younger adults' abilities to perform their usual PA routines has negative consequences for their PA. In line with this, a previous Danish study found it important for younger adults to have fixed appointments and a fixed timetable when doing PA (Pilgaard and Rask, 2016). The study also shows that younger adults to a greater extent seek stability in their everyday life when they experience uncertainties. As such, younger adults might have felt a lack of certainty during the shutdown, making it impossible to do their usual PA routines.

### Adults

Adults experienced the smallest decrease in PA (6.3% per week), and the number of adults who did PA 5 or more times per week almost doubled. Being forced to work from home or not work at all may have increased adults' flexibility and ability to do PA at different hours. Previously, adults were found to be the age group that, at most, prefer flexibility to be able to start or continue an active lifestyle (Pilgaard and Rask, 2016). Additionally, adults might not have been forced to make major adaptations to their usual PA habits during the shutdown. Adults primarily did outdoor PA alone before the shutdown, which they continued to do during the shutdown, combined with PA with their family. In fact, those living with their partner and children had the smallest decrease in minutes of PA (4.8%). This is somewhat supported by a study conducted in the UK during the shutdown. Rogers et al. (2020) find that families with school-aged children were associated with doing more intensive PA. Social support from family members and doing PA together is found to positively influence adults PA behavior (Zimmermann et al., 2008), and being “locked up” together may support doing PA in the family.

### Older Adults

Globally, half of 80+-year-old and almost a third of 60–79-year-old adults do not reach the PA recommendations (Bauman et al., 2016) and represent the least active population group in Denmark (Jensen et al., 2018). This highlights the severity of the large decrease found in this study (24.9%) and supported by other studies (Maugeri et al., 2020; Yamada et al., 2020). This age group also experienced a 71.4% increase in participants rarely or never being physically active. This suggests that many older adults went from being regularly active during the week to almost no PA. As old age is associated with functional limitations and physical health problems (de Groot et al., 2004), maintaining good physical health is important. The observed decrease in PA may be due to several factors related to older adults. Older adults were the largest group that did not gain any new PA habits during the shutdown (32.1%). This suggests that older adults may have difficulties in adapting their PA habits as they rely more on social support to change and maintain their PA habits (Lindsay Smith et al., 2017). Furthermore, older adults primarily did PA together with friends in public or in sports associations before the shutdown, which, partly, was not possible during the shutdown. This is supported by previous research arguing that social interaction is one of the main motivators for older adults to leave their home and participate in activities (Schmidt et al., 2019) and that social isolation is associated with higher inactivity (Shankar et al., 2011).

### Strength and Limitations

This study, conducted in a unique situation during the COVID-19 outbreak, offers important insights into the research field of PA behavior. This study is the first of its kind conducted in Scandinavia and the first to provides contextual data on PA behavior for different age groups. A strength of the study is that the survey is based on a standardized Danish national survey on PA behavior repeated every fourth year (Pilgaard and Rask, 2016). Although the national survey has not been validated, it has been used numerous times in the Danish context throughout the last 20 years, making it highly relevant and comparable for this specific study focusing on PA in a Danish context. It is, therefore, argued that using an international validated questionnaire for this specific study would be less feasible although using a validated questionnaire may limit measurement error (Aggio et al., 2016).

A limitation is the recruitment method, which may have limited the representativeness of the study population as the study found an overrepresentation of highly educated females and adults (30–59 years). Nevertheless, 53% of the Danish population uses Facebook every day with relatively equal distributions of gender (Statistik, 2018), and almost all other similar studies have used online recruitment methods, such as social media (Cheval et al., 2020; López-Bueno et al., 2020; Maugeri et al., 2020; Rogers et al., 2020; Smith et al., 2020). These factors suggest that Facebook may be a reliable recruitment method. On the contrary, because relevance of the survey topic is shown to influence response rates (Groves et al., 2000), using social media platforms may have caused self-selection bias with an overrepresentation of respondents who may find PA important (Lavrakas, 2008). Additionally, the survey was only conducted in Danish and, therefore, excluded a range of ethnic minority groups living in Denmark.

The study builds on retrospective and self-reported data on PA, which is valuable in obtaining people's views of their own PA behavior, but can be threatened by recall bias (Althubaiti, 2016) with great variance in type, frequency, and duration of PA. Although survey questions were on routine PA behavior, which may have minimized the recall bias (Althubaiti, 2016), the length of the recall period was “within the last 12 months,” which may be difficult for some to recall. We suggest that future studies should obtain data on citizens' PA behavior collected prior to the COVID-19 shutdown to better compare and assess any consequences of the shutdown on people's PA behavior. More so, objective (device-based) data on PA rather than self-reported data on PA prior to, during, and after the COVID-19 shutdown would be preferable. Last, because this study included Danish citizens over 15 years old, caution should be made when interpreting the results of the low-educated participants. Participants in the age category 15–29 years may not have finished a higher education yet. As such, the low-educated group may not reflect a low socioeconomic group.

## Conclusions and Perspectives

This study reveals that different subgroups of the population, already struggling to meet PA guidelines, struggled even more during the shutdown. The shutdown mostly affected teens and younger and older adults negatively as well as those with lower education and those living in rural areas. Although shutting down sports facilities and social distancing are important for preventing the spread of the virus, it can have negative consequences for people's motivation and opportunity for PA, hence exacerbating people's physical and mental health. In conclusion, the shutdown seemed to negatively impact Danish citizens PA behavior, which is concerning if this decrease in PA will persist and become the new societal norm. Efforts should, therefore, be made to help the most vulnerable groups to be physically active during and after the shutdown.

In fact, this study provided relevant information that was used in October 2020 when Denmark had to shut down again because of a second wave of COVID-19. At that time, the Danish government decided not to close fitness and other private sports centers. Organized sports in sport associations were also maintained for children and youth up to the age of 21, whereas adults over the age of 21 were allowed to do organized sports together with a maximum of 10 people. These decisions might especially help teens and younger and older adults to maintain their leisure PA during the COVID-19 pandemic and are beneficial to the health of the Danish population.

Although the study has several limitations, the importance of investigating and learning from this unique situation is argued to outweigh these limitations to spread the knowledge and act more appropriately during future pandemics. More studies are needed using objective PA data collected on all age groups as well as different minority groups, prior, and during the COVID-19 pandemic, to better assess PA change.

## Data Availability Statement

The raw data supporting the conclusions of this article will be made available by the authors, upon request.

## Ethics Statement

The study involving human participants was reviewed and approved by the Research & Innovation Organization (RIO) of University of Southern Denmark (10.975). Written informed consent from the participants' legal guardian/next of kin was not required to participate in this study in accordance with the national legislation and the institutional requirements.

## Author Contributions

CP came up with the idea for the study, helped conduct the study, and contributed to writing the paper. TS created the study design and the questionnaire, conducted the data collection and analysis, and was the main author of the paper. Both authors have commented on and approved the final manuscript.

## Conflict of Interest

The authors declare that the research was conducted in the absence of any commercial or financial relationships that could be construed as a potential conflict of interest.
